# Where Do the Poorest Go to Seek Outpatient Care in Bangladesh: Hospitals Run by Government or Microfinance Institutions?

**DOI:** 10.1371/journal.pone.0121733

**Published:** 2015-03-25

**Authors:** Yu-hwei Tseng, Mujibul Alam Khan

**Affiliations:** 1 Institute of Health Policy and Management, National Taiwan University, Taipei, Taiwan; 2 Research Initiative for Social Empowerment (RISE), Dhaka, Bangladesh

## Abstract

**Introduction:**

Health programs implemented by microfinance institutions (MFIs) aim to benefit the poor, but whether these services reach the poorest remains uncertain. This study intended to investigate the socioeconomic distribution of patients in hospitals operated by microfinance institutions (i.e. MFI hospitals) in Bangladesh and compare the differences with public hospitals to determine if the programs were consistent with their pro-poor mandate.

**Methods:**

In this cross-sectional study, we used the convenience sampling method to conduct an interviewer-assisted questionnaire survey among 347 female outpatients, with 170 in public hospitals and 177 in MFI hospitals. Independent variables were patient characteristics categorized into predisposing factors (age, education, marital status, family size), enabling factors (microcredit membership, household income) and need factors (self-rated health, perceived needs for care). We employed Generalized Estimating Equations (GEE) to evaluate how these factors contributed to MFI hospital use.

**Results:**

Use of MFI hospitals was associated with microcredit membership over 5 years (OR=2.9, p<.01), moderately poor household (OR=4.09, p<.001), non-poor household (OR=7.34, p<.01) and need for preventive care (OR=3.4, p<.01), compared with public hospitals. Combining membership and income, we found microcredit members had a higher tendency towards utilization but membership effect pertained to the non- and moderately-poor. Compared with the group who were non-members and the poorest, microcredit members who were non-poor had the highest likelihood (OR=7.46, p<.001) to visit MFI hospitals, followed by members with moderate income (OR=6.91, p<.001) and then non-members in non-poor households (OR=4.48, p<.01). Those who were members but the poorest had a negative association (OR=0.42), though not significant. Despite a higher utilization of preventive services in MFI hospitals, expenditure there was significantly higher.

**Conclusion:**

Inequity was more pronounced in MFI hospitals than public ones. MFI hospitals appeared to miss their target population. We suggest that MFIs reorganize health programs toward primary health care to make care equitable and universally accessible. This study holds practical implications for governments, development agencies and microfinance practitioners working at the grassroots level.

## Introduction

The sick among the poorest are the world’s most vulnerable people. The need to remove barriers to access healthcare is urgent, particularly in low-income countries [1,2]. While there have been attempted interventions to increase availability and accessibility to health services and products, socioeconomic differentials of the utilization have not been addressed, and the poorest segment of the population often benefitted the least [3–9]. As a result, the destitute delayed seeking health care due to their low capacity to pay [10,11] and might eventually lead to catastrophic health spending [12]. It indicates that using an equity lens in the evaluation of program design and targeting is crucial.

Working with the poorest of the poor to improve their welfare has been the professed goal of microfinance institutions (MFIs) [13,14]. Initially, microcredit practitioners were motivated to add health components to their programs when they identified unmet needs of the poor due to a lack of access and inability to afford care [15]. On the other hand, non-financial programs rewarded MFIs in terms of clients’ better ability to repay, increased client loyalty and a new income opportunity such as health financing and charging user-fees [13,16]. These two types of motivation, one from the demand side and the other from the supply side, may have a potential conflict and therefore deserve scrutiny. First, what pro-poor strategies do MFIs employ in their health programs? Second, do MFIs monitor whether the services reach their target population? And third, does the current mode of care provided best serve the interest of the poorer segment in a population?

The impact of MFIs is profound, given the huge number of people exposed to their programs. Among the 3,718 MFIs that have reported to the Microcredit Summit Campaign since 1998, 1,747 (46.99%) were based in Asia and the Pacific, and heavily concentrated in India and Bangladesh. MFIs in these two countries have reached 102 million of the poorest, of which 11.46% reside in Bangladesh, the birthplace of institutionalized microcredit programs [17]. Non-governmental organizations (NGOs) in this country have had a rich heritage of health interventions and have quickly introduced integrated projects by combining microcredit and healthcare [18–21]. As NGO-MFIs have grown larger in volume and size, some have moved beyond the realm of primary health care and started running hospitals.

Running general hospitals is a relatively new phenomenon in the evolution of MFI, and therefore evaluation of the utilization of such hospitals by different socioeconomic groups is scarce. Some studies simply pointed out a positive association between microcredit membership and utilization of health services [6,21,22]. Community-based programs using outreach health workers appears to have a positive effect on equity [3]. However, while MFIs’ primary health care programs have produced encouraging results [23], little is known about their hospital services. Decomposing data by socioeconomic strata is particularly important since MFIs uphold the signboard of reaching the poor and the poorest, which cannot be masked by mere availability. Besides the MFIs, the public sector is also working in the same direction to ensure indiscriminate access for even the most vulnerable [24,25], which has been validated in single country and multi-country research suggesting a higher likelihood of the poorest seeking care from public providers [26,27]. In this study, we concurrently investigated characteristics of patients in MFI and public hospitals. By doing so, we aimed to compare patients in two types of hospitals and present the extent to which MFI hospitals served the poorest. In light of existing literature indicating an exclusion problem in MFIs’ credit and social programs [5,28–32] we thereby hypothesized that, compared with public hospitals, MFI hospitals might serve patients from higher income groups, charge higher fees, and might be used more by microcredit beneficiaries.

It is well-documented that unequal access to care contributes to health inequalities [33]. Also a commonly held notion is that government and non-governmental actors in developing countries can be complementary in the health care delivery system [18,34–37]. Bangladesh has witnessed a slow but steady growth of NGO-MFIs engaging in hospital operation. Whether this strategy has been aligned with government policy and narrowed the equity gap requires ongoing monitoring and systematic evaluation. This paper is the first attempt to add the missing piece to the knowledge base. Understanding the impact can be translated into evidence-informed policies of governments, development agencies and large MFIs when poor-friendly initiatives are at stake.

## Materials and Methods

### Conceptual framework

**Fig 1:**
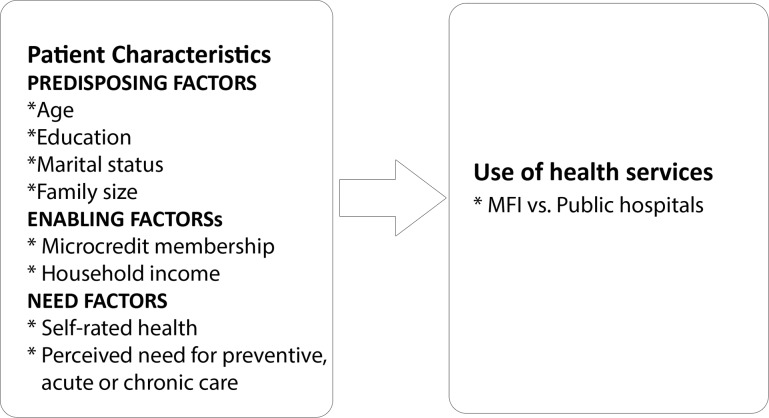
Conceptual framework for determinants of MFI hospital utilization.

Fig. 1 shows the conceptual framework for determinants of MFI hospital utilization. We employed the widely used Behavioral Model of Health Services Use developed by Ronald M. Andersen to lay out factors that influence utilization of medical care [38,39]. According to the model, usage of health services is determined primarily by population characteristics. Population characteristics can be categorized into three groups: predisposing, enabling and need. The predisposing group encompasses demographic and social structural factors. Demographic factors which suggests the likelihood of using health care are represented by age, gender and marital status. Social structural factors determines an individual’s ability to cope with problems and can be measured by one’s education, occupation and ethnicity. Enabling factors represent the means and likelihood of individuals to obtain services. These factors would include: the existence of health facilities and manpower, wealth, distance to facilities, transportation, health insurance and other context-specific measures. Last, the model investigates needs, that is, how people perceive the state of their general health, how they experience health conditions and whether they think the problems need medical intervention. Utilization takes place only when the need for care (either perceived or evaluated)—the most immediate cause of health service use—emerges. Additionally, Andersen noted that organizational factors improved our ability to explain use, so we presented consultation charges incurred after actual use, but did not include it in the model [39]. It is noteworthy that this research did not intend to repeat the same story of lower utilization of those in lower socioeconomic strata. We specifically examined if the poor-rich divide was narrower in health care facilities operated by organizations with an explicitly stipulated pro-poor mission.

### Study design and setting

This health services research used information from a cross-sectional survey of outpatients in public and MFI hospitals in Bangladesh. We set the criteria for the selection of hospitals as follows. First, they must be general hospitals, not specialized such as MCH, cancer or diabetic hospitals. Second, non-public hospitals had to be managed by microfinance institutions with a clear mandate to serve the poor. This criterion excluded non-MFI NGO or private charity hospitals. Third, for better comparison, we identified the sites where MFI and public hospitals operated as closely to each other as possible and where no private hospitals existed. Finally, taking into consideration security, time constraints and availability of transport options, we selected three MFIs located in three different districts.

Table 1 shows the basic information of the sites, hospitals and number of respondents. District A is in the rural north bordering India and the remotest of the three sites. District B is northwest of the capital Dhaka, semi-urban and well-connected by good transportation links. District C is an urban area next to the capital, an industrial and commercial zone with all forms of transport. In each selected district there is one district hospital in town providing general and specialized care, with 100, 250 and 100 beds, respectively. Healthcare outside the town is available at sub-district health complexes and community clinics. The private sector is active, but not operating full-fledged general hospitals in the study areas. The three MFIs were established in the late 1980s, and introduced microcredit during 1990–1994, basic health programs during 1996–1999, and hospitals in 2004 and 2010 on the basis of serving the poor at low cost. Though small in scale, the respective 22-, 50- and 70-bed hospitals were equipped with general physicians, X-ray, family planning, physiotherapy, immunization, ultrasonography, antenatal & postnatal care, ECG, pathology and, specialist care namely medicine, surgery, gynaecology/obstetrics, paediatrics, ophthalmology, ENT, orthopaedics, cardiology and so on.

**Table 1:** Site and hospital characteristics.

**Site characteristics** ^a^			
District code	A	B	C
Area of district in sq. km	2,841	3,424	759
# of households	479,000	866,800	671,200
Average household size	4.6	4.1	4.3
# of district hospitals	1	1	1
# of MFI that runs hospitals	1	1	1
Total # of doctors in the district	134	323	154
**Hospital characteristics**			
***Publicly run district hospital*** ^b^			
Location in district	District town	District town	District town
# of beds	100	250	100
# of patients in 2012	111,112	245,238	291,040
# of doctors (full-time)	16	47	33
# of respondents	53	46	71
***MFI-run hospital*** ^c^			
Location	District town	District town	District town
Year started	2010	2004	2010
# of beds	22	50	70
# of patients in 2012	10,800	56,445	26,212
# of doctors (full-time & part time)	22	16	24
Population coverage	n.a.	3.9 million	2.1 million
# of respondents	42	59	76
Services and facilities^d^	1–8, 10–11, 13–15,17–18	1–7, 9–10,12–18	1–6, 8–19

Sources:

^a^General information from Population & Housing Census 2011, Bangladesh Bureau of Statistics

^b^Health indicators from Health Bulletin 2013 & 2014, MoHFW Bangladesh

^c^MFI information from 2012 annual reports of MFIs

^d^Services include 1) Family planning, 2) ANC & PNC, 3) Immunization, 4) Medicine, 5) Surgery, 6) Gynaecology/obstetrics, 7) Cardiology, 8) ENT, 9) Eye, 10) Paediatrics, 11) Orthopedics,12) Physiotherapy, 13) Pathology, 14) Ultrasound, 15) ECG, 16) X-ray, 17) Pharmacy, 18) 24 hours, 19) ICU

### Data collection

We focused on how MFI hospital utilization was characterized by the socioeconomic positions of female patients. Guided by theoretical and empirical studies in South Asian countries, we developed our conceptual framework and questionnaire accordingly [3,6,7,21,40,41]. The survey collected independent variables of age, level of education, marital status, family size, microcredit membership and duration, monthly household income, self-rated health, perceived needs for preventive care (e.g. health check-up, maternal care, immunization), acute conditions (obstetric & gynaecologic treatment, fever, diarrhoea, accident/injury, acute conditions of the eye, skin or ear, cold, gastric pain, pneumonia) or chronic conditions (diabetics, hypertension, heart disease, weakness, long-term problems of the eye, skin or ear). The outcome was utilization of an MFI or public hospital. We also enquired about consultation fees as supplementary data. This item was fixed in two types of hospitals and might represent ease of access.

Sex is a typical predisposing factor. We recruited only female patients as women are usually the most vulnerable and represent 93% of all borrowers [42]. Regarding age, the minimum legal age for a woman to marry in Bangladesh is 18, but in reality one- third of women aged 20–24 were married by the age of 15 [43]. We set our inclusion threshold at 15 taking into account the need for maternal care among young married women.

Trained surveyors employed convenience sampling when conducting face-to-face interviews with patients. In MFI hospitals, interviews were completed in the waiting areas. We did not see a huge number of outpatients in MFI hospitals at the time of study. Two to three surveyors worked simultaneously so we had enough time to approach mostly all eligible patients and finish our work without missing potential respondents. There were only a few who disagreed to cooperate initially but later consented either after carefully observing our work or after being approached again by the main researcher. Interviewer screening was minimal in MFI hospitals. The waiting areas in the public hospitals, on the other hand, had a large number of patients and few seats. Interviewing under such circumstances appeared inappropriate. Arrangements were made so a corner was used by the interviewer next to the consulting room. As soon as one interview was completed, the surveyor approached the next available patient. We encountered a zero-rejection rate. Possible explanations for high level of cooperation are: one, patients considered the questioning to be part of the consultation, and two, surveyors having a chair and desk to work at may have given the appearance that it might be hospital business they should comply with. These considerations should be kept in mind for future studies. Low rejection precluded the occurrence of interviewer-related selection bias.

Ethical approval was obtained from the Research Ethics Office of National Taiwan University (NTU). The Ministry of Health & Family Welfare (MoHFW) in Bangladesh and respective MFIs approved the research. Written consent was difficult to obtain due to high illiteracy rate among women, which was 48.61% in rural and 34.05% in urban areas [44]. A written consent form was read out loud and explained to potential respondents. Upon receipt of informed verbal consent from each respondent, the investigators began the interview. Use of oral consent was approved by the Research Ethics Office of NTU and MoHFW.

### Data analysis

A total of 379 subjects were interviewed but 32 were incomplete. We then included 347 subjects in the analysis. Descriptive analyses were performed to present the distribution of socioeconomic characteristics of respondents in two types of hospitals. Bivariate associations were examined using the chi-square test. Generalized Estimating Equations (GEE) has been regarded a suitable method to analyse correlated binary responses arising from the relatedness of individuals in the same cluster [45,46]. In the present study, data collected in three different districts was likely to be clustered and correlated. Therefore, observations from the same district could not be treated as if they were independent. As typical logistic regression does not account for correlation within each area cluster, the GEE approach allowed us to properly use all data to estimate the relationship between patients’ primary determinants and health behaviour, taking into consideration the clustering effects within particular areas. By doing this we were able to make a more robust inference. Parameter estimates generated by GEE were then converted into odds ratios. We further examined the simultaneous effects of microcredit membership and income by creating new variables in the model and adjusting for confounding factors. Statistical analysis was done with SAS 9.3 (SAS Institute, Cary, NC, USA).

## Results

Table 2 displays the socioeconomic characteristics and fees paid by study subjects. There were 177 respondents in MFI hospitals and 170 in public hospitals. Mean age was 31 (SD = 13.02) and 35 (SD = 12.95) years in MFI and public hospitals, respectively. MFI hospitals had a higher percentage (67.23%) of younger patients between 15 and 30 years old than the public hospitals (44.12%). The majority of respondents were married. In MFI hospitals those with 5–9 and 10+ years of education accounted for 32.37% and 26.59%, while in public hospitals the largest subgroup was the one without any education, at 37.72%. The percentages of microcredit members in MFI and public hospitals were 31% and 26%, respectively. Microcredit borrowers in MFI hospitals had a slightly longer history of membership (4.64 years, SD = 5.01) than those in public hospitals (4.22 years, SD = 5.50). The mean family size was about 5 persons in both settings. About 70% of the respondents in MFI hospitals had a household income over 8,000 Bangladeshi Taka (US$103) per month and 5.68% reported a household income below 4,500 taka (US$58). In contrast, 46.67% of patients at public hospitals had a household income over 8,000 taka and 25.45% were below 4,500 taka. Regarding self-assessed health, after merging small-sized subgroups, 53.45% of patients in MFI hospitals reported good health (excellent, very good, good and fair versus bad health), much higher than 24.26% in public hospitals. The majority of patients (83.53%) visited public hospitals for acute conditions, but more patients visited MFI hospitals for preventive services (45.20%) than for their acute (37.85%) or chronic conditions. Consultation fees at public hospitals were mostly < 50 taka (98.82%). In MFI hospitals, the majority paid between 100 and 500 taka (74.43%). Bivariate analysis found significant differences between the two groups regarding age, marital status, education, household income, self-rated health, perceived need and cost.

**Table 2:** Patient characteristics and cost of care in MFI and public hospitals.

	MFI hospital (n = 177)	Public hospital (n = 170)	*p*
**Age (years)**			***
15–30	67.23% (119)	44.12% (75)	
≧31	32.77% (58)	55.88% (95)	
Mean age	31.19 (SD13.02)	35.27 (SD12.95)	
**Education (years)**			*
0	24.86% (43)	37.72% (63)	
1–4	16.18% (28)	16.77% (28)	
5–9	32.37% (56)	30.54% (51)	
10+	26.59% (46)	14.97% (25)	
**Marital Status**			**
Currently married^a^	93.10% (162)	84.12% (143)	
**Family size** (mean)	5.13 persons (SD 2.05)	4.99 persons (SD 1.97)	
**Microcredit membership**			
Zero membership (non-member)	69.49% (123)	74.71% (127)	
Short-term membership (<5 years)	19.21% (34)	18.24% (31)	
Long-term membership (≧5 years)	11.30% (20)	7.06% (12)	
Mean among members	4.64 (SD = 5.01)	4.22 (SD = 5.50)	
**Household Income**			***
Poorest (≦4,500 taka)	5.68% (10)	25.45% (42)	
Moderate (4,501–8,000 taka)	23.30% (41)	27.88% (46)	
Non-poor (≧8,001 taka)	71.02% (125)	46.67% (77)	
**Self-rated health**			***
Good	53.45% (93)	24.26% (41)	
Poor	46.55% (81)	75.74% (128)	
**Perceived need**			***
Preventive services	45.20% (80)	7.06% (12)	
Acute conditions	37.85% (67)	83.53% (142)	
Chronic conditions	7.34% (13)	9.41% (16)	
**Cost (consultation fee)**			***
<50 taka	17.61% (31)	98.82% (168)	
50–100 taka	6.25% (11)	0% (0)	
100–500 taka	74.43% (131)	1.18% (2)	
>500 taka	1.7% (3)	0% (0)	

*p<.05.

**p<.01.

***p<.001 (chi-square test).

^a^Others—never married, separated, divorced, widowed.

Table 3 presents the adjusted odds ratios of selected factors associated with MFI hospital utilization. After taking into account all the covariates, only the enabling and need factors had significant associations with MFI hospital use; predisposing factors did not. Compared with patients who did not participate in any microcredit program, patients with a longer history of microcredit membership were more likely to use MFI hospitals (OR = 2.90, 95% CI: 1.46–5.75 for membership more than 5 years). Income level showed a clear gradient with utilization. In comparison with public hospital respondents, moderately-poor and non-poor were 4.09 times (95% CI: 3.27–5.12) and 7.34 times (95% CI: 2.05–26.31) more likely than the poorest to go to an MFI hospital. There was an increased likelihood for those reporting good health to utilize an MFI hospital, but the association was not significant. In terms of perceived need, the odds ratio of patients using MFI hospital for preventive care was 3.4 (95% CI:1.43–8.07). However, there was a significantly negative association with the need for acute care (OR = 0.26, 95% CI: 0.08–0.90).

**Table 3:** Adjusted odds ratios (and 95% confidence intervals) of factors associated with MFI-hospital utilization.

	Adjusted OR	95% CI
***Predisposing factors***		
**Age** (ref. ≧31 years)		
Young (15–30 years)	1.28	0.62–2.62
**Education** (ref. 0~4 years)		
≧5 years	1.03	0.53–2.04
**Marriage** (ref. unmarried)		
Married	1.32	0.65–2.69
**Family size**	1.02	0.83–1.24
***Enabling factors***		
**Microcredit membership** (ref. zero membership)		
Short-term membership (<5 years)	1.54	0.60–3.94
Long-term membership (≧5 years)	2.90**	1.46–5.75
**Income level** (ref. poorest)		
Moderately poor (4,501–8,000 taka)	4.09***	3.27–5.12
Non-poor (≧8,001 taka)	7.34**	2.05–26.31
***Need factors***		
**Self-rated health** (ref. poor health)		
Good health	1.78	0.84–3.74
**Perceived need** (ref. chronic care)		
Preventive care	3.40**	1.43–8.07
Acute care	0.26*	0.08–0.90

*p<.05.

**p<.01.

***p<.001.

Table 4 provides the adjusted odds ratios of new variables combining income level and microcredit membership that predicted MFI hospital use. Besides a gradient along income levels in both member and non-member subgroups, the former had a higher tendency towards utilization. Compared with the group who were non-members and the poorest, microcredit members who were non-poor had the highest likelihood (OR = 7.46, p<.001) to visit MFI hospitals, followed by members with moderate income (OR = 6.91, p<.001) and then non-members in non-poor households (OR = 4.48, p<.01). Those who were members but the poorest had a negative association (OR = 0.42), though not significant.

**Table 4:** Adjusted odds ratios of combined factors to predict MFI hospital utilization.

	Membership with MFI			
Variables^a^	Member		Non-member	
Income level	OR	(95% CI)	OR	(95% CI)
Non-poor	7.46***	(2.51–22.14)	4.48**	(1.82–11.07)
Moderately poor	6.91***	(3.68–12.96)	1.91	(0.99–3.68)
Poorest	0.42	(0.12–1.52)	1^b^	(ref.)

*p<.05.

**p<.01.

***p<.001.

^a^This model adjusted for age, marital status, education, family size, need and self-rated health.

^b^Reference group: those with the poorest household income and without membership in any microfinance institution.

## Discussion

### Did MFI services reach their target population?

Compared with outpatients in public settings, those in MFI hospitals tended to be younger, married, better educated, wealthier, seeking preventive care and spending more. After adjusting for known confounders, the poor-rich difference remained substantial. Unequal use of facility-based services by different economic classes has been noted in previous research [3,4,7,8,47]. However, inequity was more pronounced in MFI-affiliated hospitals than public ones. MFI hospitals appeared to miss their target population.

### Did MFI hospitals employ pro-poor strategies in their health programs?

As hypothesized, MFI hospitals were more likely to serve higher income groups and charge significantly higher fees. As a result, poor patients were less likely to visit MFI hospitals and were unable to afford a visit. The findings were not unexpected because researchers have noted that outside the public sector, not only private companies, healthcare facilities run by NGOs and MFIs also facilitated use-inequality by high service charge [6,7,48,49]. In poor people’s own words, the NGO healthcare was meant for the rich [49]. This opinion coincided with the patient profile mapped by this research.

Income disparities played the greatest role in the unequal use of MFI hospitals. This research reiterated the fact that financial constraint is a major barrier for the poorest to use health care [50,51]. The ability to pay and the price of service are two sides of the same coin. The choices for the poorest segment of the population were systematically restricted by the pricing schemes of MFI hospitals. Studies [7,9] showed that when services were provided free of charge, poor people visited NGO facilities more than public ones, which was probably due to advantages in the NGO sector, such as closer relationships, a strong reputation at the grassroots, motivated staff and better quality of service. However, when NGO-MFI hospitals charged patients at a much higher rate than public ones fewer poor patients used them, as observed in our study. User fees at MFI hospitals did not appear to be poor-friendly.

### Credit membership and utilization

Our findings supported the third hypothesis that MFI hospitals were used more by microcredit borrowers. Length of participation in credit programs exhibited a significant impact on a woman’s choice of provider. This finding was consistent with existing literature in which microcredit membership was associated with an array of positive outcomes, i.e. service utilization [6,21], health behaviours [52,53] and maternal knowledge [54]. The dose-effect relationship was indicated in previous explorations between duration of membership and outcomes like poverty reduction or health knowledge [55–58], as a result of borrowers’ enhanced capabilities over time [59]. We also documented a dose effect as well as a combined effect of income and membership on utilization. Nevertheless, the membership effect was limited to moderately- and non-poor, and probably to town dwellers. The negative association among the poorest can be interpreted in two ways. First, income can play a decisive role in patients’ choice making, which meant that borrowers who were the poorest, *still* could not afford care in an MFI hospital. And secondly, people living in towns were more often better off than those in rural areas. Most of the borrowers live and work in rural areas, which limits their inclination to pay for more expensive services. Moreover, the cost of traveling and wages lost may also play into their decision as to which hospital to go to for care. Hospital care made available by MFIs did not seem to significantly increase accessibility among the poorest microcredit borrowers.

### Whose interest did the current mode of provision serve?

Following the exclusion of the poorest from credit programs, a similar tendency to marginalize the poorest patients from facility-based health programs was noted in this study. The reason for the former was to reduce the risk of bad debt [60,61] and the underlying cause could be the same for both credit and social programs. Cull et al. (2007) summed it up as a trade-off between financial sustainability and outreach to the poor [62]. Provision of secondary hospital care is a costly investment, therefore to maintain hospital operations the management needs to take a business approach such as reducing risks and cost, increasing revenue, improving productivity, and enhancing utilization [63]. Providing preventive care for a higher fee in urban areas and targeting healthier patients of higher socioeconomic status with a sense to invest in health could well fulfil these goals. In the current study, not only did MFI hospital patients report better health, they also reported higher levels of household income and need for preventive care, a similar phenomenon noted in developed societies [64,65]. As healthcare evolves towards a business model and the provision of care becomes dependent upon a patient’s ability to pay, the poorest are further marginalized. Ahmed and colleagues (2006) expressed the same concern by noting that if NGOs rely on cost recovery through user-fees they will inevitably stray from the goal of service to the poor [49].

### Limitations

A major limitation of this study is that we only assessed a few socio-demographic and economic correlates. We could not exhaust all the factors at the individual and organizational levels. Quality and other hospital characteristics undoubtedly mattered, but they were beyond the scope of this research which primarily pinpointed the socioeconomic position of MFI hospital users. We excluded hospital size due to a high degree of multicollinearity with hospital ownership. Secondly, the data were from a small sample in selected towns. Therefore, the findings pertain to the hospitals at the time of the interview. Thirdly, we recognized that our simple measurement of income might be inaccurate. Underreporting of household income in developing countries is commonplace [66–68]; it is seen as a systematic error and difficult to deal with. The underreporting of agricultural income in Bangladesh and elsewhere has been regarded as worse than any other sector. However, the notion that underreporting by the rich is more prevalent than among the poor [66–68] has given us confidence that the gap identified in our research would be even wider, if we did obtain accurate income information. Despite these limitations, this study may be the first to identify and gauge the magnitude of the socioeconomic divide in MFI hospital use. It highlighted the necessity of further evaluation regarding the effectiveness of MFI hospital programs in serving the poorest patients.

## Conclusions

This study examined whether the rich-poor gap was effectively narrowed in health care facilities established to provide care for the poorest. It found marked inequality in utilization and income disparities contributed most. The poorest people, borrowers and non-borrowers alike, did not benefit much from MFI hospital initiatives. As health inequalities worsen in developing countries, the implications are profound. Participation in credit programs had different impacts on households of different socioeconomic situations. The limitations of using microcredit as a platform to deliver public goods or strengthen health systems have been illustrated in this research. We offer two suggestions. First, rather than operating hospitals in urban areas, MFIs may reorganize health programs around the principles of primary health care, namely, bringing affordable care as close as possible to where people live and work [69]. MFIs have demonstrated great strength in community-based disease prevention and health promotion [3,34] and this might be the areas where MFIs can better contribute to poverty reduction. Mere availability of services does *not* guarantee equitable or affordable access. The importance of primary health care and the fair distribution of this care cannot be overemphasized [70,71]. To sustain equity in health care utilization and uplift the poorest, we believe this would be a wise strategy. Second, regular monitoring is important to recognize the degree to which the poor benefit from targeted programs. The policy-making processes require essential information from routine examinations as well as research. It would ensure that health and other development programs stay focused on the organization’s mission. This holds true for policy makers in both government and NGO sectors. In the development, implementation and evaluation of health programs, concerned authorities and NGOs must always take note of the inequality gap and examine what component widens the gap and makes people more vulnerable. This is the key to holding MFIs accountable and responsive to all stakeholders.

## Supporting Information

S1 Fig Conceptual framework for determinants of MFI hospital utilization. (TIF)

S1 Table Site and hospital characteristics. ^a^ General information from Population & Housing Census 2011, Bangladesh Bureau of Statistics
^b^ Health indicators from Health Bulletin 2013 & 2014, MoHFW Bangladesh
^c^ MFI information from 2012 annual reports of MFIs
^d^ Services include 1) Family planning, 2) ANC & PNC, 3) Immunization, 4) Medicine, 5) Surgery, 6) Gynaecology/obstetrics, 7) Cardiology, 8) ENT, 9) Eye, 10) Paediatrics, 11) Orthopedics,12) Physiotherapy, 13) Pathology, 14) Ultrasound, 15) ECG, 16) X-ray, 17) Pharmacy, 18) 24 hours, 19) ICU
(DOCX)

S2 Table Patient characteristics and cost of care in MFI and public hospitals. *p<.05; **p<.01; ***p<.001 (chi-square test).
^a^ Others—never married, separated, divorced, widowed.
(DOCX)

S3 Table Adjusted odds ratios (and 95% confidence intervals) of factors associated with MFI-hospital utilization. *p<.05; **p<.01; ***p<.001
(DOCX)

S4 Table Adjusted odds ratios of combined factors to predict MFI hospital utilization. *p<.05; **p<.01; ***p<.001
^a^ This model adjusted for age, marital status, education, family size, need and self-rated health.
^b^ Reference group: those with the poorest household income and without membership in any microfinance institution.
(DOCX)
