# Supplementary figures and images for: Where Do the Poorest Go to Seek Outpatient Care in Bangladesh: Hospitals Run by Government or Microfinance Institutions?
Source: PLoS One. 2015 Mar 25;10(3):e0121733. doi: 10.1371/journal.pone.0121733 (PMC4373946; doi:10.1371/journal.pone.0121733)

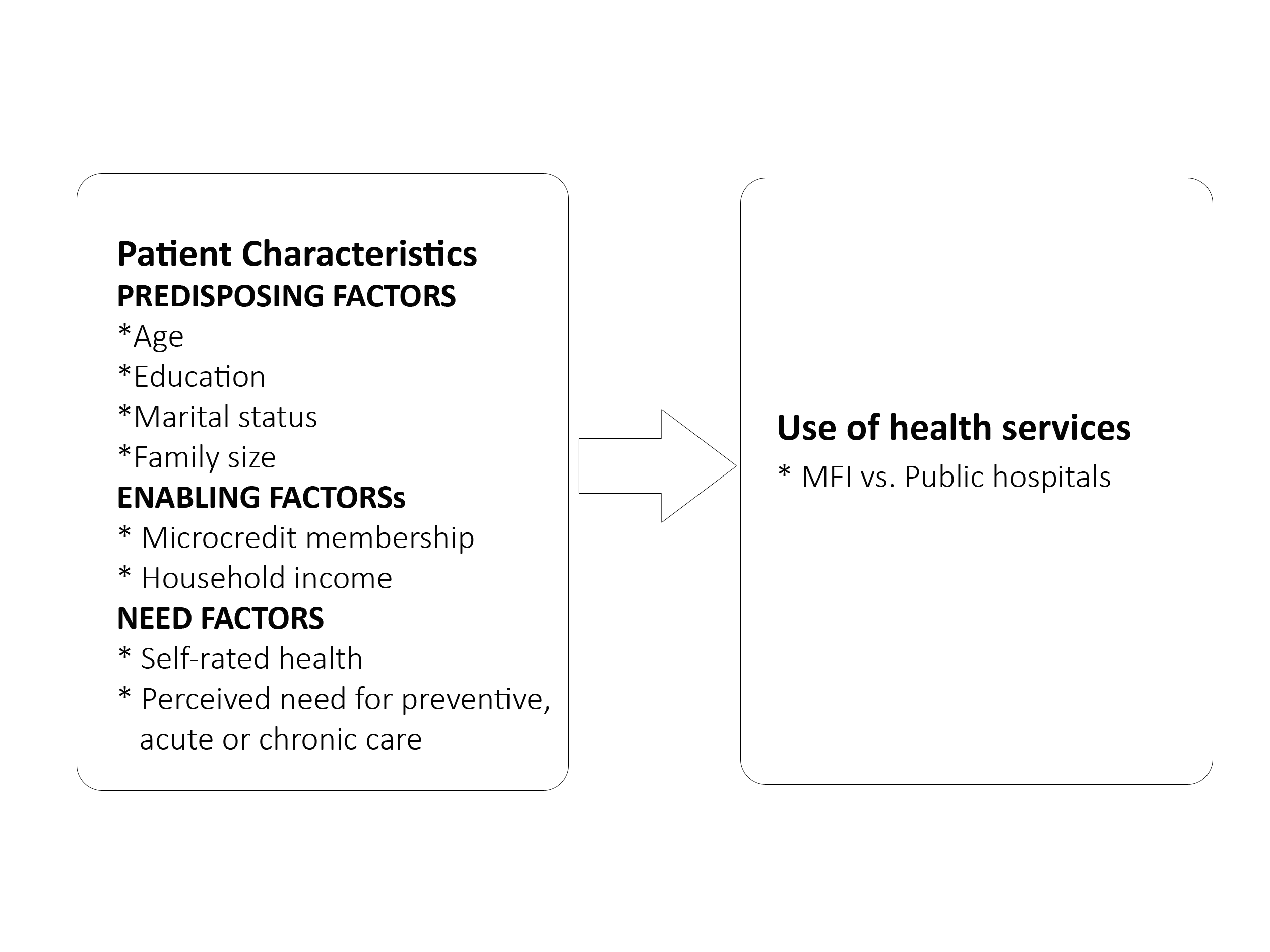

Supplement: S1 Fig — (TIF) [file pone.0121733.s001.tif]
