# Supplementary material for: Where Do the Poorest Go to Seek Outpatient Care in Bangladesh: Hospitals Run by Government or Microfinance Institutions?
Source: PLoS One. 2015 Mar 25;10(3):e0121733. doi: 10.1371/journal.pone.0121733 (PMC4373946; doi:10.1371/journal.pone.0121733)
Supplement: S1 Table — a General information from Population & Housing Census 2011, Bangladesh Bureau of Statistics b Health indicators from Health Bulletin 2013 & 2014, MoHFW Bangladesh c MFI information from 2012 annual reports of MFIs d Services include 1) Family planning, 2) ANC & PNC, 3) Immunization, 4) Medicine, 5) Surgery, 6) Gynaecology/obstetrics, 7) Cardiology, 8) ENT, 9) Eye, 10) Paediatrics, 11) Orthopedics,12) Physiotherapy, 13) Pathology, 14) Ultrasound, 15) ECG, 16) X-ray, 17) Pharmacy, 18) 24 hours, 19) ICU (DOCX) [file pone.0121733.s002.docx]

**Table 1. Site and hospital characteristics**

| **Site characteristics**^a^ | | | |
| --- | --- | --- | --- |
| District code | A | B | C |
| Area of district in sq. km | 2,841 | 3,424 | 759 |
| # of households | 479,000 | 866,800 | 671,200 |
| Average household size | 4.6 | 4.1 | 4.3 |
| # of district hospitals | 1 | 1 | 1 |
| # of MFI that runs hospitals | 1 | 1 | 1 |
| Total # of doctors in the district | 134 | 323 | 154 |
| **Hospital characteristics** | | | |
| ***Publicly run district hospital***^b^ | | | |
| Location in district | District town | District town | District town |
| # of beds | 100 | 250 | 100 |
| # of patients in 2012 | 111,112 | 245,238 | 291,040 |
| # of doctors (full-time) | 16 | 47 | 33 |
| # of respondents | 53 | 46 | 71 |
| ***MFI-run hospital***^c^ | | | |
| Location | District town | District town | District town |
| Year started | 2010 | 2004 | 2010 |
| # of beds | 22 | 50 | 70 |
| # of patients in 2012 | 10,800 | 56,445 | 26,212 |
| # of doctors (full-time & part time) | 22 | 16 | 24 |
| Population coverage | n.a. | 3.9 million | 2.1 million |
| # of respondents | 42 | 59 | 76 |
| Services and facilities^d^ | 1-8, 10-11, 13-15,17-18 | 1-7, 9-10,12-18 | 1-6, 8-19 |
| Sources: |  |  |  |

^a^General information from Population & Housing Census 2011, Bangladesh Bureau of Statistics

^b^Health indicators from Health Bulletin 2013 & 2014, MoHFW Bangladesh

^c^MFI information from 2012 annual reports of MFIs

^d^Services include 1) Family planning, 2) ANC & PNC, 3) Immunization, 4) Medicine, 5) Surgery, 6) Gynaecology/obstetrics, 7) Cardiology, 8) ENT, 9 ) Eye, 10) Paediatrics, 11) Orthopedics,12) Physiotherapy, 13) Pathology, 14) Ultrasound, 15) ECG, 16) X-ray, 17) Pharmacy, 18) 24 hours, 19) ICU
