# Supplementary material for: Where Do the Poorest Go to Seek Outpatient Care in Bangladesh: Hospitals Run by Government or Microfinance Institutions?
Source: PLoS One. 2015 Mar 25;10(3):e0121733. doi: 10.1371/journal.pone.0121733 (PMC4373946; doi:10.1371/journal.pone.0121733)
Supplement: S2 Table — *p<.05; **p<.01; ***p<.001 (chi-square test). a Others—never married, separated, divorced, widowed. (DOCX) [file pone.0121733.s003.docx]

**Table 2. Patient characteristics and cost of care in MFI and public hospitals**

|  | **MFI hospital (n=177)** | **Public hospital (n=170)** | ***p*** |
| --- | --- | --- | --- |
| **Age (years)** |  |  | *** |
| 15 – 30 | 67.23% (119) | 44.12% (75) |  |
| ≧31 | 32.77% (58) | 55.88% (95) |  |
| Mean age | 31.19 (SD13.02) | 35.27 (SD12.95) |  |
| **Education (years)** |  |  | * |
| 0 | 24.86% (43) | 37.72% (63) |  |
| 1-4 | 16.18% (28) | 16.77% (28) |  |
| 5-9 | 32.37% (56) | 30.54% (51) |  |
| 10+ | 26.59% (46) | 14.97% (25) |  |
| **Marital Status** |  |  | ** |
| Currently married^a^ | 93.10% (162) | 84.12% (143) |  |
| **Family size** (mean) | 5.13 persons (SD 2.05) | 4.99 persons (SD 1.97) |  |
| **Microcredit membership** |  |  |  |
| Zero membership (non-member) | 69.49% (123) | 74.71% (127) |  |
| Short-term membership (＜5 years) | 19.21% (34) | 18.24% (31) |  |
| Long-term membership (≧5 years) | 11.30% (20) | 7.06% (12) |  |
| Mean among members | 4.64 (SD=5.01) | 4.22 (SD=5.50) |  |
| **Household Income** |  |  | *** |
| Poorest (≦4,500 taka) | 5.68% (10) | 25.45% (42) |  |
| Moderate (4,501-8,000 taka) | 23.30% (41) | 27.88% (46) |  |
| Non-poor (≧8,001 taka) | 71.02% (125) | 46.67% (77) |  |
| **Self-rated health** |  |  | *** |
| Good | 53.45% (93) | 24.26% (41) |  |
| Poor | 46.55% (81) | 75.74% (128) |  |
| **Perceived need** |  |  | *** |
| Preventive services | 45.20% (80) | 7.06% (12) |  |
| Acute conditions | 37.85% (67) | 83.53% (142) |  |
| Chronic conditions | 7.34% (13) | 9.41% (16) |  |
| **Cost (consultation fee)** |  |  | *** |
| <50 taka | 17.61% (31) | 98.82% (168) |  |
| 50-100 taka | 6.25% (11) | 0% (0) |  |
| 100-500 taka | 74.43% (131) | 1.18% (2) |  |
| >500 taka | 1.7% (3) | 0% (0) |  |

*p<.05; **p<.01; ***p<.001 (chi-square test).

^a^Others - never married, separated, divorced, widowed.
