# Supplementary material for: Where Do the Poorest Go to Seek Outpatient Care in Bangladesh: Hospitals Run by Government or Microfinance Institutions?
Source: PLoS One. 2015 Mar 25;10(3):e0121733. doi: 10.1371/journal.pone.0121733 (PMC4373946; doi:10.1371/journal.pone.0121733)
Supplement: S3 Table — *p<.05; **p<.01; ***p<.001 (DOCX) [file pone.0121733.s004.docx]

**Table 3. Adjusted odds ratios (and 95% confidence intervals) of factors associated with MFI-hospital utilization**

|  | **Adjusted OR** | **95% CI** | | |
| --- | --- | --- | --- | --- |
| ***Predisposing factors*** |  |  |  |  |
| **Age** (ref. ≧31 years) |  |  |  |  |
| Young (15-30 years) | 1.28 | 0.62 | － | 2.62 |
| **Education** (ref. 0~4 years) |  |  |  |  |
| ≧5 years | 1.03 | 0.53 | － | 2.04 |
| **Marriage** (ref. unmarried) |  |  |  |  |
| Married | 1.32 | 0.65 | － | 2.69 |
| **Family size** | 1.02 | 0.83 | － | 1.24 |
| ***Enabling factors*** |  |  |  |  |
| **Microcredit membership** (ref. zero membership) | |  |  |  |
| Short-term membership (<5 years) | 1.54 | 0.60 | － | 3.94 |
| Long-term membership (≧5 years ) | 2.90** | 1.46 | － | 5.75 |
| **Income level** (ref. poorest) |  |  |  |  |
| Moderately poor (4,501-8,000 taka) | 4.09*** | 3.27 | － | 5.12 |
| Non-poor (≧8,001 taka) | 7.34** | 2.05 | － | 26.31 |
| ***Need factors*** |  |  |  |  |
| **Self-rated health** (ref. poor health) |  |  |  |  |
| Good health | 1.78 | 0.84 | － | 3.74 |
| **Perceived need** (ref. chronic care) |  |  |  |  |
| Preventive care | 3.40** | 1.43 | － | 8.07 |
| Acute care | 0.26* | 0.08 | － | 0.90 |

*p<.05; **p<.01; ***p<.001
