# Supplementary material for: Where Do the Poorest Go to Seek Outpatient Care in Bangladesh: Hospitals Run by Government or Microfinance Institutions?
Source: PLoS One. 2015 Mar 25;10(3):e0121733. doi: 10.1371/journal.pone.0121733 (PMC4373946; doi:10.1371/journal.pone.0121733)
Supplement: S4 Table — *p<.05; **p<.01; ***p<.001 a This model adjusted for age, marital status, education, family size, need and self-rated health. b Reference group: those with the poorest household income and without membership in any microfinance institution. (DOCX) [file pone.0121733.s005.docx]

**Table 4. Adjusted odds ratios of combined factors to predict MFI hospital utilization**

|  | **Membership with MFI** | | | | |
| --- | --- | --- | --- | --- | --- |
| **Variables^a^** | **Member** | |  | **Non-member** | |
| **Income level** | **OR** | **(95% CI)** |  | **OR** | **(95% CI)** |
| Non-poor | 7.46*** | (2.51-22.14) |  | 4.48** | (1.82-11.07) |
| Moderately poor | 6.91*** | (3.68-12.96) |  | 1.91 | (0.99-3.68) |
| Poorest | 0.42 | (0.12-1.52) |  | 1^b^ | (ref.) |
|  | | | | | |

*p<.05; **p<.01; ***p<.001

^a^This model adjusted for age, marital status, education, family size, need and self-rated he**a**lth.

^b^Reference group: those with the poorest household income and without membership in any microfinance institution.
